# Urine gamma-synuclein as a biomarker for the diagnosis of bladder cancer

**DOI:** 10.18632/oncotarget.9468

**Published:** 2016-05-19

**Authors:** Caiyun Liu, Bingbing Shi, Chonghua Hao, Qinghai Wang, Qiang Lv, Nianzeng Xing, Jianzhong Shou, Like Qu, Yanning Gao, Chao Qin, Jiyu Zhao, Chengchao Shou

**Affiliations:** ^1^ Key Laboratory of Carcinogenesis and Translational Research (Ministry of Education) and Molecular Biology, Peking University Cancer Hospital & Institute, Beijing, China; ^2^ Department of Biochemistry & Molecular Biology, Peking University Cancer Hospital & Institute, Beijing, China; ^3^ Department of Urology, Peking Union Medical College Hospital, Chinese Academy of Medical Sciences and Peking Union Medical College, Beijing, China; ^4^ Department of Clinical Laboratory, Shanxi Provincial People's Hospital, Taiyuan, China; ^5^ Department of Kidney Transplantation, The Affiliated Hospital of Qingdao University, Qingdao, China; ^6^ Department of Urology, First Affiliated Hospital of Nanjing Medical University, Nanjing, China; ^7^ Department of Urology, Beijing Chaoyang Hospital, Capital Medical University, Beijing, China; ^8^ Department of Urology, Cancer Hospital, Chinese Academy of Medical Sciences, Beijing, China; ^9^ Department of Etiology and Carcinogenesis, Cancer Institute & Hospital, Chinese Academy of Medical Sciences, Beijing, China

**Keywords:** urine, gamma-synuclein, bladder cancer, diagnosis, prognosis

## Abstract

Gamma-synuclein (SNCG) is secreted from tumor cells and elevated in the urine of bladder cancer (BCa) patients, however, the diagnostic and prognostic values of urine SNCG for BCa remain unknown. Here, we used enzyme immunoassay and western blotting to measure urine SNCG levels. Patients with BCa or other urological diseases and healthy controls were enrolled at four Chinese hospitals from April 2010 to November 2014. Diagnostic performance was evaluated by analyzing the area under receiver operating characteristic curves (AUROCs). The AUROC was 0.903 ± 0.019 (95% confidence interval [CI], 0.867 - 0.940) for the test and 0.929 ± 0.015 (95% CI, 0.901 - 0.958) for the validation cohort. The optimal cutoff value yielded sensitivities of 68.4%, 62.4% and specificities of 97.4%, 97.8% for the test and validation cohort, respectively. Urine SNCG levels were decreased after tumor resection, but were higher in BCa patients with recurrence than those without (*P* = 0.001). The urine SNCG levels in patients with urological benign diseases were significantly lower than BCa patients (all *P* < 0.05) but higher than healthy controls (all *P* < 0.05). Hematuria did not interfere with the SNCG detection by spiking urine specimens with whole blood. Compared with a nuclear-matrix-protein-22 assay in an additional cohort excluding hematuria, SNCG showed a similar sensitivity and higher specificity. In summary, our results demonstrated that urine SNCG can discriminate BCa from urinary diseases, and is a useful prognosticator of postsurgical recurrence.

## INTRODUCTION

Bladder cancer (BCa) is one of the most expensive tumors to treat because of the continued invasive and costly surveillance required due to its high propensity for recurrence [1]. Although approximately 75% of newly diagnosed BCa is noninvasive (i.e., early stage) [2], it frequently progresses to muscle-invasive BCa (MIBC), and the 1- and 5-year recurrence rates for nonmuscle-invasive BCa (NMIBC) range from 15% to 61% and 31% to 78%, respectively [3]. Cystoscopy and voided urinary cytology are currently the primary methods used for detecting BCa. However, cystoscopy is invasive, uncomfortable, and costly, and causes urinary tract infection at a rate of 4.5% [4]. Urinary cytology is less invasive and more specific, but has limited sensitivity, especially for low-grade disease [5, 6]. Thus, a sensitive, noninvasive test is needed for BCa diagnosis and postsurgical surveillance.

Urine-based biomarkers, such as bladder tumor antigen (BTA) and nuclear matrix protein-22 (NMP22), may be more sensitive than urine cytology [5, 7], making them useful for detecting low-grade tumors, but noncancerous conditions, including hematuria, can interfere with the detection of BTA and NMP22 [8, 9]. Consequently, none of these biomarkers is recommended for BCa detection [10]. However, diagnostic assays based on the detection of multiple biomarkers are often more accurate than those based on a single biomarker. Therefore, the detection of new biomarkers either alone or in combination with existing biomarkers might improve the diagnostic accuracy of current BCa tests [11].

**Figure 1:**
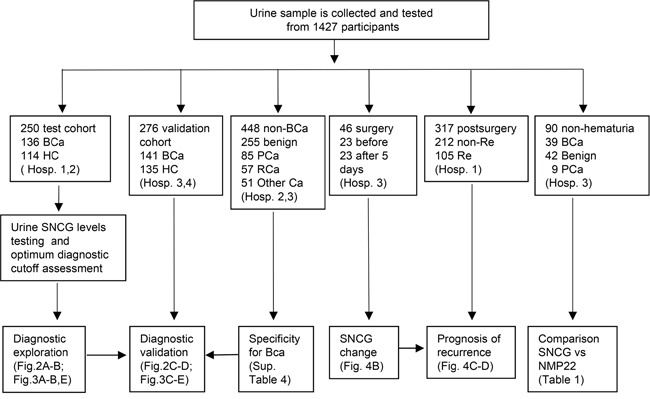
Schematic illustration of the study BCa, Bladder cancer; HC, healthy control; Benign, nonmalignant urological diseases; PCa, prostate cancer; RCa, renal carcinoma; Other cancer (nonurological malignancies listed in Supplementary Table 4); non-Re, non-recurrence; Re, recurrence; Hosp. 1, Cancer Hospital of the Chinese Academy of Medical Sciences; Hosp. 2, Beijing Chaoyang Hospital; Hosp. 3, Peking Union Medical College Hospital; Hosp. 4, Shanxi Provincial People's Hospital.

Synucleins are small, soluble proteins expressed primarily in neural tissue and in certain tumors. The synuclein family includes α-synuclein, β-synuclein, and γ-synuclein (SNCG). SNCG is overexpressed in various tumor tissues and predict adverse outcomes in breast [12, 13], colon [14, 15], and pancreatic [16] cancer patients. SNCG decreases rigidity of microtubule bundles caused by paclitaxel [17], stimulates membrane-initiated estrogen signaling by chaperoning estrogen receptor (ER)-alpha36 [18], activates mTOR signaling [19], insulin-like growth factor I (IGF-I)/IGF-IR signaling [20], and mitogen-activated protein kinases (MAPK) signaling pathways [21]. SNCG secreted from tumor cells by unconventional secretion pathway [22] and elevated serum levels of SNCG are found in pancreatic [23], gastrointestinal, esophageal, and colorectal [24, 25] cancers. The diagnostic value of combined SNCG with other multiple markers have measured with western blotting [26], ELISA [27], or western blotting and ELISA [28], but they all lacked an independent validation for BCa or without urine SNCG levels from urological diseases [27] or postsurgical BCa patients [26, 28]. In the present study, we assessed the specificity, precision, reliability of a newly-developed SNCG ELISA. The diagnostic capability and postsurgical surveillance for BCa were assessed in detecting human urine SNCG in a multi-center study with a validation cohort. The effect of hematuria on the SNCG detection and comparison with NMP-22 were also included in the study (Figure 1).

## RESULTS

### Diagnostic performance of urine SNCG quantification

The precision, accuracy, and reliability of the urine SNCG ELISA were established as the follow. The mean coefficient of variation (CV) of 2.9% ± 1.9% for intra-assay variation and 8.0% ± 2.1% for inter-assay variation (Supplementary Table 1) demonstrated high reproducibility. The spike recovery in human urine ranged from 84.6% ± 6.5% to 101.5% ± 6.0% (Supplementary Table 2) and the dilution recovery of SNCG in urine from 97.4% ± 9.7% to 110.5% ± 10.8% (Supplementary Table 3).

**Figure 2:**
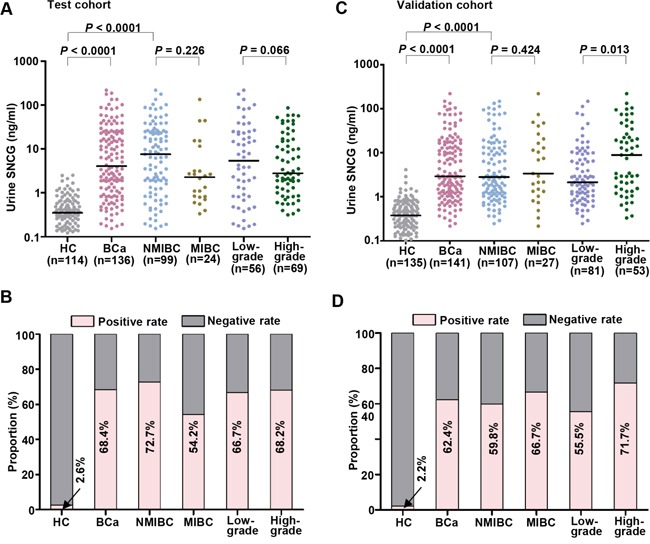
Scatter plot of urine SNCG in the test and validation cohorts Distribution of urine SNCG levels in the test cohort **A.** and validation cohort **C.** SNCG-positive rates in all BCa, NMIBC/MIBC, low-grade/high-grade BCa in the test cohort **B.** and validation cohort **D.** Black horizontal lines are median values. Gamma-synuclein, SNCG; HC, healthy control; BCa, Bladder cancer; NMIBC/MIBC, nonmuscle/muscle invasive bladder cancer.

The detail clarification for various sub-studies in the study was illustrated in Figure 1. The distributions of urine SNCG levels and the urine SNCG positive and negative rates in the test and validation cohorts were shown in Figure 2A to 2D. The median of urine SNCG was 4.07 ng/mL (range, 0.15–671.40 ng/mL) in BCa patients (n=136), 0.35 ng/mL (range, 0.01–2.51 ng/mL) in controls (n=114) in the test cohort, and 2.93 ng/mL (range, 0.22–218.79 ng/mL, n=141) and 0.38 ng/mL (range, 0.01–4.11 ng/mL, n=135), respectively, in the validation cohort.

**Figure 3:**
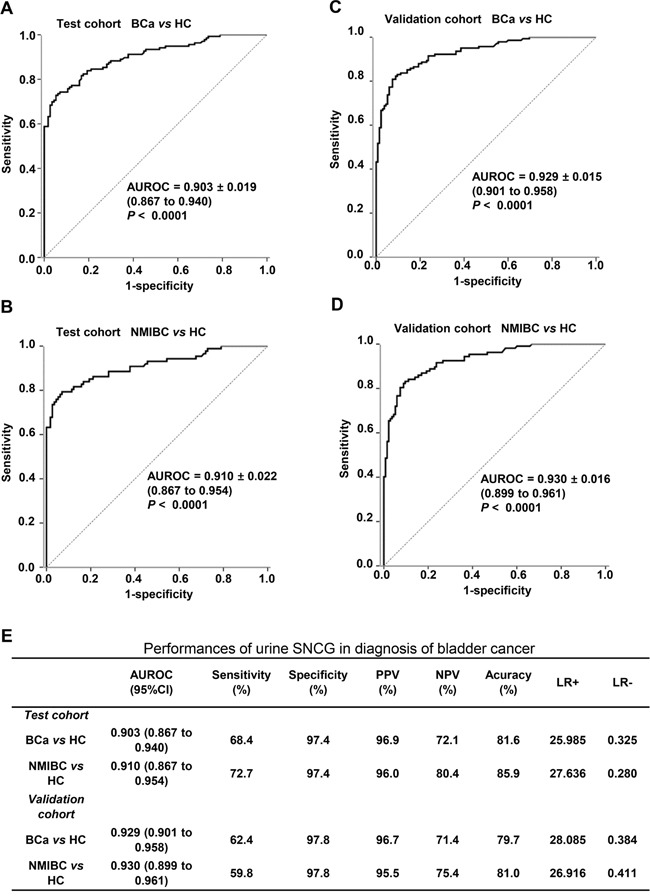
Diagnostic performance of the SNCG ELISA for all BCa and NMIBC patients versus healthy controls The ROC curves of SNCG for all BCa patients **A, C.** and NMIBC patients **B, D.** versus healthy control in the test cohort (A, B) and validation cohort (C, D). Performance characteristics of urine SNCG for the diagnosis of BCa in both the test and validation cohorts **E.** The specificity and sensitivity for urine SNCG detection was determined using an optimal cutoff value of 1.874 ng/mL. The area under the ROC and 95% confidence interval (bracketed) for SNCG are shown. Gamma-synuclein, SNCG; ELISA, enzyme-linked immunosorbent assay; BCa, bladder cancer; NMIBC, nonmuscle invasive bladder cancer; ROC, receiver operating characteristic.

In the test cohort, the AUROC was 0.903 for BCa versus control and 0.910 for early-stage BCa (i.e., NMIBC) versus control (Figure 3A and 3B, respectively). In the validation cohort, the AUROC was 0.929 for BCa versus control and 0.930 for early-stage BCa versus control (Figure 3C and 3D, respectively). The optimal cutoff value determined by Youden index for differentiating the BCa patients from controls in the test cohort was 1.874 ng/mL, resulting in a sensitivity of 68.4% and a specificity of 97.4% in the test cohort, 72.7% and 97.4% for early-stage BCa /NMIBC (Figure 3E). Similar results were observed in the validation cohort using the same cutoff (Figure 3E). The positive and negative rates of sub-BCa groups were also shown in Figure 2B, 2D. These results demonstrated the ability of SNCG to diagnose BCa even in early-stage/NMIBC disease, suggesting urine SNCG might be useful for BCa screening in a high risk population.

### Prognostic value of postsurgical urine SNCG level

**Figure 4:**
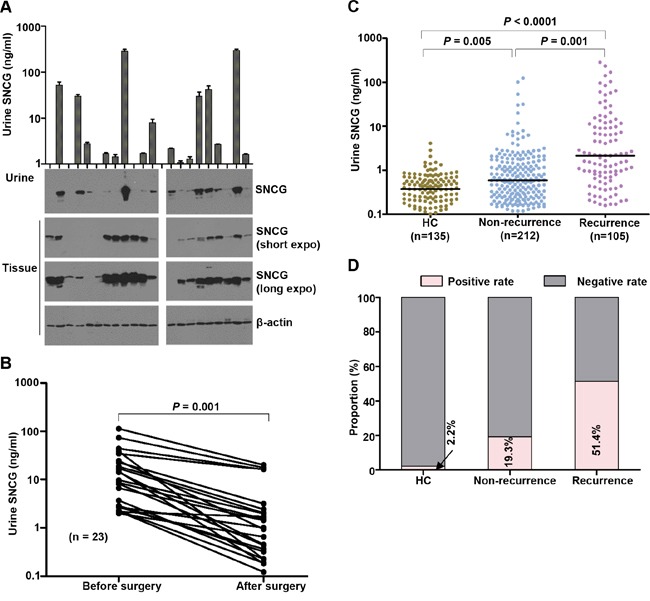
SNCG expression in paired urine and tissue and urine SNCG measurement for BCa patients before and after surgical tumor resection, and those with a history of BCa Gamma-synuclein (SNCG) expression in paired urine and tissue specimens from BCa patients **A.** Urine (4 μL) from BCa patients mixed with 1 μL 5× sample buffer in each lane. Total tissue protein (5 μg) per lane was loaded, and β-actin was used as an internal control. One representative western blotting from three independent experiments is shown. Urine SNCG concentrations in paired urine samples collected before and 5 days after resection **B.** Scatter plot of urine SNCG in patients with a history of BCa and healthy controls in the test cohort **C.** The SNCG positive rates in BCa patients with and without recurrence **D.** Black horizontal lines are median values. Gamma-synuclein, SNCG; HC, healthy control; BCa, bladder cancer.

We assessed the SNCG levels in paired tumor tissue and urine samples from 21 cases representing different stages of BCa progression using western blotting. High concordance (76.2%, 16/21) was observed between urine and tissue SNCG, except for three cases (lane 1, 8, 10) with high tissue but low urine SNCG level and two cases (lane 5 and 13) with high urine but low tissue expression (Figure 4A). Heterogeneity in tumor expression and high-level expression in vascular endothelium cells may have contributed to these results [24]. The expression profiles of SNCG in tumor tissues suggest that bladder cancer tumors are the source of urine SNCG.

As shown in Figure 4B, the median concentration of urine SNCG was 9.63 ng/mL (range, 1.98–113.42 ng/mL) before and 1.0 ng/mL (range, 0.12–20.14 ng/mL) after surgery (*n* = 23, *P* < 0.0001). The median postsurgical urine SNCG level was higher in patients who experienced a subsequent recurrence (median, 2.14 ng/mL; range, 0.16–286.10 ng/mL, n=105) than in those who did not (median, 0.59 ng/mL; range, 0.12–124.50 ng/mL, n=212; *P* = 0.001; Figure 4C). Using the same cutoff value as the test and validation cohorts, the positive rates were 51.43% and 19.34% for patients who did and did not suffer recurrence, respectively (Figure 4D). The AUROC was 0.718 for recurrent BCa versus nonrecurrent BCa, higher than that of 0.6293 for SNCG of Soukup et al's results [27].

### Follow-up of healthy controls with positive urine SNCG

Elevated urine SNCG levels were found in six healthy controls, 3 in the test cohort and 3 in the validation cohort (Figure 2). We followed up these individuals for 12 to 30 months. None of the subjects developed bladder cancer symptoms or had suspicious ultrasonographic examination. However, one was dead with hepatic metastases of lymphoma after 24 months of urine SNCG detection. Two were diagnosed as nephritis and one as prostatomegaly after 12 months of urine SNCG detection. The other two have no evidence of bladder neoplastic disease or urological diseases.

### False-positive rates

The overall false positive rate was 20.5% (92/448). The urine SNCG concentrations and false positive rates in patients with other types of malignancies and nonmalignant urological conditions were significantly lower than those in BCa patients (all *P* < 0.05), but were higher than those in the healthy controls (all *P* < 0.05) (Supplementary Table 4 and Supplementary Figure 1).

### Comparison of SNCG and NMP22 as diagnostic factors

The NMP22 BladderChek point-of-care test has been used for BCa diagnosis and surveillance [29, 30], however hematuria was shown to cause false-positive results in NMP-22 detection [8]. So, we collected urine samples excluding those with hematuria and compared the diagnostic performances of the NMP22 BladderChek with that of SNCG ELISA. The basic characteristics of patients with BCa (*n* = 39), benign diseases (*n* = 42), and prostate cancer (*n* = 9) were shown in Supplementary Table 5. The NMP22 test demonstrated slightly higher sensitivity (56.4% vs 46.2%, *P* = 0.344) and significantly lower specificity (51.0% vs 74.5%, *P* = 0.008) than the SNCG ELISA (Table 1), and the positive predictive value (PPV), negative predictive value (NPV), and accuracy were also shown in Table 1. The quantification of both biomarkers increased the sensitivity to 64.1% (Table 1). The false positive rates for the NMP22 test and SNCG ELISA were 52.4% (22/42) and 31.0% (13/42) in patients with benign urological diseases and 33.3% (3/9) and 0% (0/9) in patients with prostate cancer, respectively.

**Table 1:** Comparison of the detection of NMP22 and Gamma-Synuclein for the diagnosis of bladder cancer

	Sensitivity (%)	Specificity (%)	PPV (%)	NPV (%)	Accuracy (%)
NMP22	56.4	51.0	46.8	60.5	53.3
Gamma-Synuclein	46.2	74.5	58.1	64.4	62.2
*P*-value^a^	0.344	0.008	−	−	−
Combination	64.1	54.9	47.2	62.2	53.3

a NMP22 compared to gamma-synuclein.

Abbreviations: NMP22, nuclear matrix protein-22; PPV, positive predictive value; NPV, negative predictive value.

### Effect of hematuria

**Figure 5:**
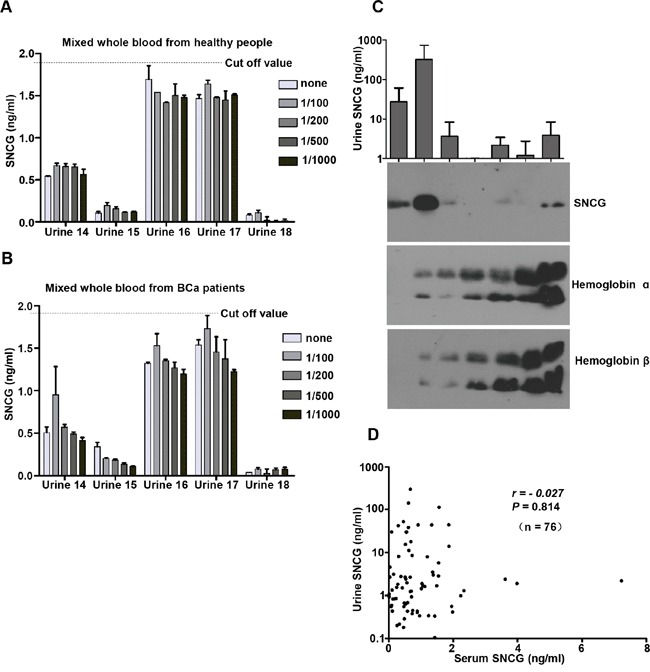
Effect of hematuria on SNCG ELISA performance The performance of the SNCG ELISA was evaluated in an experimental model of hematuria **A, B.** and SNCG, hemoglobin-α, and hemoglobin-β in urine samples with various degrees of hematuria (lane 2-7) and one without hematuria (lane 1) as control were detected by western blotting **C.** Even very high levels of whole blood (1:100 dilution) did not affect urine SNCG detection (*P* > 0.05). Error bars indicate standard deviation. The dotted lines represent the threshold value of urine SNCG. Urine (4 μL) was mixed with 1 μL of 5 x nonreducing sample buffer in each lane. Correlation of matched urine and serum SNCG levels **D.** Results are expressed as the mean ± standard deviation of three independent experiments performed in duplicate. Gamma-synuclein, SNCG; ELISA, enzyme-linked immunosorbent assay.

To investigate whether the urine SNCG level was affected by hematuria severity, we added diluted whole blood from control subjects (Figure 5A, *P* = 0.969) or BCa patients (Figure 5B, *P* = 0.932) to urine samples and compared the urine SNCG changes before and after adding whole blood. As shown in Figure 5A and 5B, no significant effect on the SNCG ELISA detection was found. Moreover, we randomly selected one sample without hematuria (Figure 5C, lanes 1) and six urine samples with different degrees of hematuria (Figure 5C, lanes 2-7), and examined the levels of Hb-α, Hb-β, and SNCG by western blotting. The urine SNCG concentration did not correlate with the levels of Hb-α or Hb-β. Also, urine SNCG levels quantified by the ELISA and western blotting yielded fully consistent results (Figure 4A, upper panel, Figure 5C, upper panel), suggesting the reliability of SNCG detection. No correlation between paired serum and urine SNCG levels (*r* = −0.027, *P* = 0.814, n = 76, Figure 5D) further confirmed that blood did not affect the ELISA of SNCG detection. The level of SNCG was significantly higher in urine (mean ± SD, 15.94 ± 48.76 ng/mL, range, 0.03-297.34 ng/mL) than in serum (0.92 ± 1.05 ng/mL, range, 0.01-7.23 ng/mL, *P* = 0.009). Serum SNCG levels can not predict bladder cancer in those patients without hematuria, there is no significant difference between BCa patients and healthy controls (*P* = 0.618).

## DISCUSSION

High correlation of SNCG levels between paired urine and tissue suggests BCa tissue as a source for urine SNCG, which supported that SNCG actively secreted from cancer cells by unconventional secretion pathway [22]. Consistent urine SNCG levels obtained by SNCG ELISA and western blotting ensured the reliability of urine SNCG detection.

We measured human urine SNCG levels using a one-step sandwich enzyme immunoassay within an hour incubation of urine samples with a pair of anti-SNCG monoclonal antibodies (MAb). SNCA (α-synuclein) and SNCB (β-synuclein), sharing 54% and 56% amino acid sequence identity with SNCG, respectively, cannot be detected by the SNCG ELISA [24]. Reagents used in the assay, including 7 serially diluted SNCG standards, are ready to use and stored at 4°C with stability for at least six months. The ELISA kits used by Soukup et al [27] and Kumar et al [28] included multiple steps with biotin-labelled polyclonal anti-human SNCG antibody and streptavidin-HRP system during 2.5 h [27] or 3.5 h [28] incubations. User need to reconstitute the lyophilized standard with diluent buffer as the stock solution and dilute it to different concentrations prior to the assay, which may easily give rise to intra- and inter-variations. Some reagents should be stored at −20°C while the others should be at 4°C with 3 months stability [28].

Hematuria severely affected the specificity of the BTAsta [9] and NMP22 [8, 31] detection and increased the false-positive results. We used the same methods of spiking urine specimens with whole blood as described previously [8, 9, 31], and found that hematuria did not affect the specificity of the SNCG ELISA detection. This finding was further supported by the absence of a correlation between the urine levels of SNCG and Hb-α or Hb-β. Kumar et al reported hematuria had no effect on urine biomarker detections by quantifying the western blotting intensity of the biomarkers in blood and urine samples and normalized them to human serum albumin (HSA) values in each respective sample [28]. Urine HSA level varied considerably and was higher in patients with chronic diseases than that from the BCa patients [28]. So the effect of hematuria on the five urine biomarker detections remained puzzled.

We found that urine SNCG levels in patients with other cancers and nonmalignant disorders were higher than those in the healthy controls, but were much lower than in the BCa patients. The total false-positive rate was 20.5% (92/448), and nonmalignant urological diseases was 23.9% (61/255), in accordance with Iwaki et al' results of the false-positive rate of 22% (52/230) in non-bladder cancer patients [26]. We have compared the diagnostic value of urine SNCG with NMP22 BladderChek in an additional cohort excluding hematuria. Fifteen BCa patients were positive and 14 BCa patients were negative for both biomarkers. Seven were NMP-22-positive and SNCG-negative, and 3 were NMP-22-negative and SNCG-positive. These results indicate that the sensitivities of NMP-22 and SNCG were similar and there was a little complementary with each other. The optimal urine biomarker(s) that can complement the limits of SNCG is under investigation, which will provide a more sensitive and specific diagnostic tool for BCa than the currently available methods.

## MATERIALS AND METHODS

### Study population

In the prospective study, we enrolled patients with signs and symptoms of urinary tract malignancy, such as hematuria and dysuria, and suspicious ultrasonographic results on a consecutive basis within a 4-year period. All patients provided urine samples and the SNCG level was conducted in a blinded manner. The status of newly diagnosed patients was decided based on symptoms of urinary tract malignancy, ultrasonographic results, and chief complaints of patients. The finally diagnostic result was based on cystoscopy and histopathological examination. Patients with multiple urological diseases or undefined pathological status were excluded. The test cohort was recruited at the Cancer Hospital of the Chinese Academy of Medical Sciences and Beijing Chaoyang Hospital from April 2010 to December 2012, and consisted of 136 newly diagnosed BCa patients and 114 control subjects who had been deemed healthy after undergoing a physical examination. The validation cohort was recruited at Peking Union Medical College Hospital and Shanxi Provincial People's Hospital from October 2012 to November 2014, and consisted of 141 newly diagnosed BCa patients and 135 healthy control subjects. Patients who had a history of bladder cancer, including 105 with and 212 without recurrent BCa by cystoscopy, were also included. We also recruited 255 patients with various nonmalignant urological conditions and 193 patients with non-BCa malignancies (Supplementary Table 4). We also collected urine samples from 23 primary BCa patients, excluding those treated with intravesical perfusion, before and 5 days after tumor resection to evaluate the postsurgical prognostic value of urine SNCG. The detail distribution of the participants used in the study was shown in Figure 1. All of the BCa cases were histopathologically confirmed by a qualified pathologist. Tumor staging and grading were performed according to criteria established by the World Health Organization. Informed written consent was obtained before samples were collected, and our study protocols were approved by the Institutional Review Board at each institution.

### Urine SNCG detection

Urine samples were collected, aliquoted, and stored at −80°C prior to cystoscopy. The level of urine SNCG was analyzed in a blinded fashion by two researchers using a one-step sandwich ELISA. Upon completion of the study, we decoded the patient data, and compared the participants' urine SNCG levels with their clinicopathological status to evaluate the diagnostic performance of the SNCG ELISA.

The level of urine SNCG was quantified using the ELISA and an SNCG standard was purified from MCF7-SNCG cells, as described previously [24]. A 50-μL aliquot of each urine sample or 50 μL of an SNCG standard solution was combined with 50 μL horse radish peroxidase-labelled anti-SNCG monoclonal antibody (MAb) no. 1 in assay buffer (10 mM phosphate-buffered saline, 0.1% Tween-20, and 10% fetal calf serum) in microtiter plates coated with another anti-SNCG MAb no. 42. The plate was incubated for 1 h with shaking. After four washes, a substrate solution was added, and the plates were incubated for 15 min at room temperature before adding 100 μL of 2 N sulfuric acid. The absorbance of the well contents was measured at 450 nm (product) and 630 nm (reference). Replicate analyses were performed and the urine SNCG concentrations were extrapolated from the standard curve. Samples with an absorbance greater than that of the 10 ng/mL standard were diluted in assay buffer, and measured again. SNCG levels in some urine samples were valued using both the ELISA and western blotting to validate the ELISA-based quantification of SNCG.

### The effect of hematuria on urine SNCG detection

To assess whether the detection of SNCG was affected by hematuria, SNCG levels were determined in each urine sample (Urine samples from 14 to 18, Figure 5) before and after adding different concentrations of whole blood from healthy controls (*n* = 20) and BCa patients (*n* = 13). Additional details of the ELISA analysis are included in the Supplementary Materials.

### Western blotting

The level of urine SNCG was analyzed by western blotting with the mouse anti-SNCG MAb no. 1 used in the ELISA above [22, 24]. Rabbit anti-hemoglobin (Hb)-α and anti-Hb-β polyclonal antibodies (Santa Cruz Biotechnology, Dallas, TX) were used as primary antibodies and goat anti-rabbit or anti-mouse IgG conjugated with horse radish peroxidase was used as secondary antibodies (Santa Cruz Biotechnology). The details of the western blotting procedure are included in the Supplementary Materials.

### Statistical analysis

All variables were tested for normality using the Shapiro-Wilk test. The correlation between urine SNCG concentrations and clinicopathological characteristics was analyzed using a Pearson χ^2^ test or Fisher exact test. Inter-group variation was evaluated using *t*-test or paired *t*-test. The diagnostic performance of the SNCG ELISA was evaluated by analyzing the area under receiver operator characteristic curves (AUROCs). Sensitivity and specificity were evaluated at an optimal cutoff using the Youden index. Accuracy, positive and negative predictive values, and likelihood ratios were also calculated using this threshold. Sample size estimated to determine the performance of the SNCG detection was based on One-Sample Sensitivity and Specificity Power Analysis. A total sample size of 228 (which includes 114 subjects with the disease) achieves 90% power to detect a change in sensitivity from 0.5 to 0.65 using a two-sided binomial test and 100% power to detect a change in specificity from 0.5 to 0.97 using a two-sided binomial test. The target significance level is 0.05. The actual significance level achieved by the sensitivity test is 0.0487 and achieved by the specificity test is 0.0487. All statistical analyses were performed using the SPSS, Version 19 (IBM, Armonk, NY). A two-tailed probability of *P* < 0.05 was considered statistically significant.

## SUPPLEMENTARY FIGURES AND TABLES



## References

[1] Sievert KD, Amend B, Nagele U, Schilling D, Bedke J, Horstmann M, Hennenlotter J, Kruck S, Stenzl A (2009). Economic aspects of bladder cancer: what are the benefits and costs?. World J Urol.

[2] Burger M, Catto JW, Dalbagni G, Grossman HB, Herr H, Karakiewicz P, Kassouf W, Kiemeney LA, La Vecchia C, Shariat S, Lotan Y (2013). Epidemiology and risk factors of urothelial bladder cancer. Eur Urol.

[3] Sylvester RJ, van der Meijden AP, Oosterlinck W, Witjes JA, Bouffioux C, Denis L, Newling DW, Kurth K (2006). Predicting recurrence and progression in individual patients with stage Ta T1 bladder cancer using EORTC risk tables: a combined analysis of 2596 patients from seven EORTC trials. Eur Urol.

[4] Almallah YZ, Rennie CD, Stone J, Lancashire MJ (2000). Urinary tract infection and patient satisfaction after flexible cystoscopy and urodynamic evaluation. Urology.

[5] Brown FM (2000). Urine cytology. It is still the gold standard for screening? Urol Clin North Am.

[6] Konety BR, Getzenberg RH (2001). Urine based markers of urological malignancy. J Urol.

[7] Mungan NA, Vriesema JL, Thomas CM, Kiemeney LA, Witjes JA (2000). Urinary bladder cancer test: a new urinary tumor marker in the follow-up of superficial bladder cancer. Urology.

[8] Miyake M, Goodison S, Giacoia EG, Rizwani W, Ross S, Rosser CJ (2012). Influencing factors on the NMP-22 urine assay: an experimental model. BMC Urol.

[9] Oge O, Kozaci D, Gemalmaz H (2002). The BTA stat test is nonspecific for hematuria: an experimental hematuria model. J Urol.

[10] Kamat AM, Hegarty PK, Gee JR, Clark PE, Svatek RS, Hegarty N, Shariat SF, Xylinas E, Schmitz-Dräger BJ, Lotan Y, Jenkins LC, Droller M, van Rhijn BW, Karakiewicz PI (2013). ICUD-EAU International Consultation on Bladder Cancer 2012: Screening, diagnosis, and molecular markers. Eur Urol.

[11] Gaston KE, Grossman HB (2010). Proteomic assays for the detection of urothelial cancer. Methods Mol Biol.

[12] Guo J, Shou C, Meng L, Jiang B, Dong B, Yao L, Xie Y, Zhang J, Chen Y, Budman DR, Shi YE (2007). Neuronal protein synuclein gamma predicts poor clinical outcome in breast cancer. Int J Cancer.

[13] Wu K, Quan Z, Weng Z, Li F, Zhang Y, Yao X, Chen Y, Budman D, Goldberg ID, Shi YE (2007). Expression of neuronal protein synuclein gamma gene as a novel marker for breast cancer prognosis. Breast Cancer Res Treat.

[14] Liu C, Dong B, Lu A, Qu L, Xing X, Meng L, Wu J, Eric Shi Y, Shou C (2010). Synuclein gamma predicts poor clinical outcome in colon cancer with normal levels of carcinoembryonic antigen. BMC Cancer.

[15] Liu C, Qu L, Dong B, Xing X, Ren T, Zeng Y, Jiang B, Meng L, Wu J, Shou C (2012). Combined phenotype of 4 markers improves prognostic value of patients with colon cancer. Am J Med Sci.

[16] Hibi T, Mori T, Fukuma M, Yamazaki K, Hashiguchi A, Yamada T, Tanabe M, Aiura K, Kawakami T, Ogiwara A, Kosuge T, Kitajima M, Kitagawa Y (2009). Synuclein-gamma is closely involved in perineural invasion and distant metastasis in mouse models and is a novel prognostic factor in pancreatic cancer. Clin Cancer Res.

[17] Zhang H, Kouadio A, Cartledge D, Godwin AK (2011). Role of gamma-synuclein in microtubule regulation. Exp Cell Res.

[18] Shi YE, Chen Y, Dackour R, Potters L, Wang S, Ding Q, Wang Z, Liu YE (2010). Synuclein gamma stimulates membrane-initiated estrogen signaling by chaperoning estrogen receptor (ER)-alpha36, a variant of ER-alpha. Am J Pathol.

[19] Liang W, Miao S, Zhang B, He S, Shou C, Manivel P, Krishna R, Chen Y, Shi YE (2015). Synuclein γ protects Akt and mTOR and renders tumor resistance to Hsp90 disruption. Oncogene.

[20] Li M, Yin Y, Hua H, Sun X, Luo T, Wang J, Jiang Y (2010). The reciprocal regulation of gamma-synuclein and IGF-I receptor expression creates a circuit that modulates IGF-I signaling. J Biol Chem.

[21] Pan ZZ, Bruening W, Giasson BI, Lee VM, Godwin AK (2002). Gamma-synuclein promotes cancer cell survival and inhibits stress- and chemotherapy drug-induced apoptosis by modulating MAPK pathways. J Biol Chem.

[22] Liu C, Qu L, Lian S, Tian Z, Zhao C, Meng L, Shou C (2014). Unconventional secretion of synuclein-gamma promotes tumor cell invasion. FEBS J.

[23] Li Z, Sclabas GM, Peng B, Hess KR, Abbruzzese JL, Evans DB, Chiao PJ (2004). Overexpression of synuclein-gamma in pancreatic adenocarcinoma. Cancer.

[24] Liu C, Guo J, Qu L, Bing D, Meng L, Wu J, Shou C (2008). Applications of novel monoclonal antibodies specific for synuclein-gamma in evaluating its levels in sera and cancer tissues from colorectal cancer patients. Cancer Lett.

[25] Liu C, Ma H, Qu L, Wu J, Meng L, Shou C (2012). Elevated serum synuclein-gamma in patients with gastrointestinal and esophageal carcinomas. Hepatogastroenterology.

[26] Iwaki H, Kageyama S, Isono T, Wakabayashi Y, Okada Y, Yoshimura K, Terai A, Arai Y, Iwamura H, Kawakita M, Yoshiki T (2004). Diagnostic potential in bladder cancer of a panel of tumor markers (calreticulin, gamma -synuclein, and catechol-o-methyltransferase) identified by proteomic analysis. Cancer Sci.

[27] Soukup V, Kalousová M, Capoun O, Sobotka R, Breyl Z, Pešl M, Zima T, Hanuš T (2015). Panel of Urinary Diagnostic Markers for Non-Invasive Detection of Primary and Recurrent Urothelial Urinary Bladder Carcinoma. Urol Int.

[28] Kumar P, Nandi S, Tan TZ, Ler SG, Chia KS, Lim WY, Bütow Z, Vordos D, De la Taille A, Al-Haddawi M, Raida M, Beyer B, Ricci E (2015). Highly sensitive and specific novel biomarkers for the diagnosis of transitional bladder carcinoma. Oncotarget.

[29] Mowatt G, Zhu S, Kilonzo M, Boachie C, Fraser C, Griffiths TR, N'Dow J, Nabi G, Cook J, Vale L (2010). Systematic review of the clinical effectiveness and cost-effectiveness of photodynamic diagnosis and urine biomarkers (FISH, ImmunoCyt, NMP22) and cytology for the detection and follow-up of bladder cancer. Health Technol Assess.

[30] Xylinas E, Kluth LA, Rieken M, Karakiewicz PI, Lotan Y, Shariat SF (2014). Urine markers for detection and surveillance of bladder cancer. Urol Oncol.

[31] Atsu N, Ekici S, Oge OO, Ergen A, Hasçelik G, Ozen H (2002). False-positive results of the NMP22 test due to hematuria. J Urol.

