# Supplementary material for: Urine gamma-synuclein as a biomarker for the diagnosis of bladder cancer
Source: Oncotarget. 2016 May 19;7(28):43432–41. doi: 10.18632/oncotarget.9468 (PMC5190035; doi:10.18632/oncotarget.9468)
Supplement: Supplementary file 1 [file oncotarget-07-43432-s001.pdf]

SUPPLEMENTARY DATA

Western blotting

For the analysis of SNCG protein expression in bladder cancer tumors, cell lysates were prepared by homogenizing tumor tissues in a buffer containing 10 mM pH 8.0 PBS, 1% Triton x-100 (PBST), and 0.25% sodium deoxycholate and protease inhibitor cocktail (Roche Applied Science, Indianapolis, IN). Cell lysates or urine samples were analyzed by sodium dodecyl sulfate-polyacrylamide gel electrophoresis in a 15% acrylamide gel. The resolved protein bands electrophoretically transferred to nitrocellulose membranes (GE Healthcare, Waukesha, WI), and blocked for 2 h in 4% nonfat dry milk in PBST. The blots were probed using the antibodies indicated. The protein bands were developed using the Super Signal Kit (GE Healthcare), according to the manufacturer's instructions.

Paired urine and tumor samples were analyzed to compare the levels of SNCG expression. The tissue samples were obtained immediately after surgical resection and the serum samples, urine samples were also stored at -80°C. Serum SNCG was detected by the ELISA method, SNCG in tumor tissue was detected by western blotting, and SNCG in urine was valued using both the ELISA and western blotting to validate the ELISA-based quantification of SNCG. Serum samples were diluted 5-fold in assay diluent, and urine was not diluted for the ELISA.

Evaluation of the SNCG ELISA

Prior to the sample analyses, the interassay variability was assessed based on the analysis of 10 plates using three different SNCG standards and urine samples with low, medium, and high SNCG levels. The assay precision was satisfactory, with a mean coefficient of variation (CV) of 2.9% ± 1.9% for intra-assay variation and 8.0% ± 2.1% for interassay variation (Supplementary Table 1), thereby demonstrating high reproducibility. The spike recovery in human urine ranged from 84.6% ± 6.5% to 101.5% ± 6.0% (Supplementary Table 2). The dilution recovery of SNCG from urine ranged from 97.4% ± 9.7% to 110.5% ± 10.8% (Supplementary Table 3).

We assessed the performance of the sandwich ELISA by evaluating the calibration curve, detection limit, recovery, specificity, precision, and dilution linearity. A series of standards (10 to 0.16 ng/mL) were prepared by diluting purified SNCG in assay diluent. Quality control samples containing three different concentrations of SNCG were analyzed in each microtiter plate. We performed an analytical evaluation of the ELISA-based detection SNCG in urine.

The lowest limit of detection was determined from 20 buffer samples, and calculated as the mean plus three standard deviations. Three different concentrations of SNCG standard and three urine samples (Urine 1 to 3) were analyzed 10 times to determine the intra-assay and interassay variation. Replicate samples were assayed, and the assay variability was expressed as % CV = (SD/mean) × 100. Urine samples (Urine 4 to 8) were spiked with different amounts of purified SNCG for recovery analysis based on the differences between the expected and observed values. For the linearity analysis, 5 urine samples (Urine 9 to 13), which had high concentrations of SNCG, were diluted 1:2, 1:4, and 1:8, and the percentage of difference between the expected and observed values was calculated.

Supplementary Table S1: Precision of the quantification of Gamma-Synuclein by the enzyme-linked immunosorbent assay

	Coefficient of variation (%)	
	Intra-assay (*n* = 10)	Interassay (*n* = 10)
Standard SNCG (10 ng/mL)	2.231	5.523
Standard SNCG (5 ng/mL)	1.873	5.668
Standard SNCG (2.5 ng/mL)	6.389	10.302
Urine 1	0.749	9.652
Urine 2	3.064	7.234
Urine 3	3.196	9.476
Mean ± standard deviation	2.9 ± 1.9	8.0 ± 2.1

Supplementary Table S2: Recovery of Gamma-Synuclein from spiked urine samples

Amount added	Urine 4	Urine 5	Urine 6	Urine 7	Urine 8	Mean ± SD
2 ng/mL	112.0%	89.8%	109.6%	84.9%	102.4%	99.7 ± 12.0%
5 ng/mL	107.4%	106.9%	102.5%	95.6%	94.9%	101.5 ± 6.0%
10 ng/mL	95.8%	83.9%	79.2%	83.3%	81.0%	84.6 ± 6.5%

Supplementary Table S3: Dilution linearity of urine samples with high levels of Gamma-Synuclein

Dilution factor	Urine 9	Urine 10	Urine 11	Urine 12	Urine 13
None	100%	100%	100%	100%	100%
x2	103.2%	101.9%	116.6%	103.8%	108.4%
x4	106.5%	99.1%	103.8%	104.8%	93.9%
x8	101.0%	93.5%	103.4%	122.9%	89.9%
Mean ± SD	103.6 ± 2.8%	98.2 ± 4.3%	107.9 ± 7.6%	110.5 ± 10.8%	97.4 ± 9.7%

Supplementary Table S4: False-positive rates and urine levels of Gamma-Synuclein in patients without bladder cancer

	Cases	False positives (%)	Median (ng/mL)	Range (ng/mL)	*P*-value[a]	*P*-value[b]
Nonmalignant urological diseases[c]	255	23.9% (61/255)	0.692	0.07-51.29	< 0.0001	< 0.0001
Prostate cancer	85	17.6% (15/85)	0.936	0.12-24.52	< 0.0001	0.003
Renal carcinoma	57	14.0% (8/57)	0.535	0.08-20.44	< 0.0001	0.017
Other malignancies[d]	51	15.7% (8/51)	0.827	0.13-3.65	< 0.0001	< 0.0001

Note. [a]Compared with bladder cancer. [b]Compared with healthy controls. [c]Included patients with urolithiasis (n=79), urinary tract infections and inflammatory conditions (n=66), prostatic hyperplasia (n=48), benign cyst and nephropathy (benign lesions or cysts of the kidney), benign adrenal diseases, and refractory voiding (n=62). [d]Included 16 lung cancer, 25 gastrointestinal cancer, 10 gynecologic cancer cases.

Supplementary Table S5: Basic characteristics of patients

Characteristics	Cases	Mean age and range (y)	Men/ Women
Bladder cancer	39	63.8 (35 to 92)	27/12
Benign diseases	42	46.9 (17 to 85)	32/10
Prostate cancer	9	66.6 (61 to 71)	9

Note. Benign diseases included 9 cystitis cases, 11 prostatitis cases, 11 nephropathy cases, 9 urinary tract infection cases, one ureteric calculus case, and 1 papilloma case.

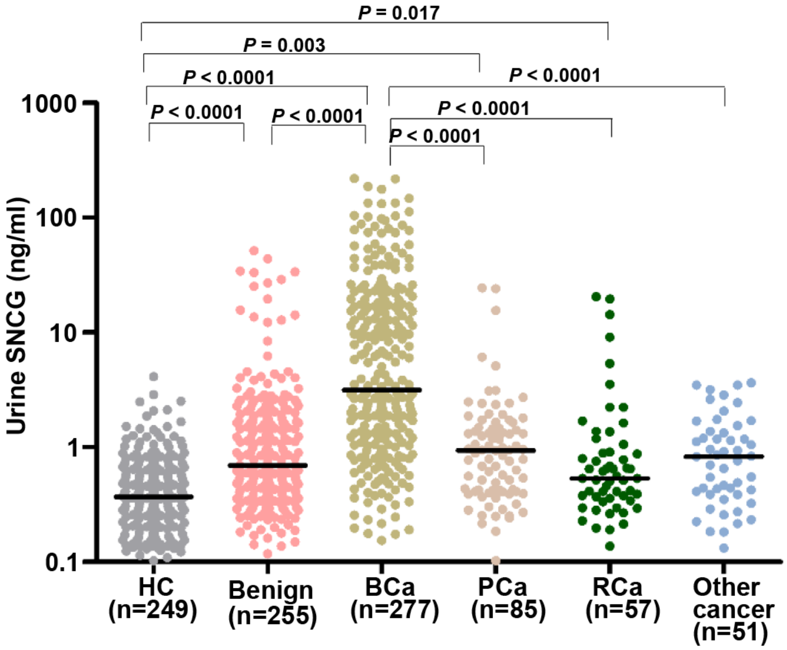

Supplementary Figure S1: Scatter plot of gamma-synuclein (SNCG) in urine based on diagnosis. Black horizontal lines are median values. BCa, Bladder cancer; Benign, nonmalignant urological diseases; Pca, prostate cancer; Rca, renal carcinoma; Other cancer (nonurological malignancies listed in Supplementary Table 4); HC, healthy control.
